# Antibiotic-resistance *Staphylococcus aureus* isolated from cow’s milk in the Hawassa area, South Ethiopia

**DOI:** 10.1186/1476-0711-11-26

**Published:** 2012-09-17

**Authors:** Deresse Daka, Solomon G/silassie, Dawit Yihdego

**Affiliations:** 1 Hawassa University College of Medicine and Health sciences, Hawassa, Ethiopia; 2 Addis Ababa University College of Medicine, Addis Ababa, Ethiopia

**Keywords:** Antibiotics, S. aureus, Milk

## Abstract

**Background:**

Quarter milk samples from cows were examined to determine the prevalence of *Staphylococcus aureus* (SA) and different antibiotic resistant pattern were determined in a cross-sectional study design.

**Objective:**

The objective of this study was to isolate *Staphylococcus aureus* from samples of cow’s milk obtained from Hawassa area and to determine their antibiotic susceptibility patterns.

**Method:**

A total of 160 milk (CCP1-CCP5) samples were collected and screened for the presence of *S. aureus*. Gram staining, oxidase, catalase, DNase, haemolysis and coagulase tests were employed for bacterial identification.

**Results:**

All the samples were contaminated with *S. aureus*. A total of 78 *S. aureus* isolates were obtained during this study. The levels of contamination with *S. aureus* were higher in milk obtained from CCP1, CCP2, CCP3, CCP4 and CCP5 at Hawassa area farms (18.0%, 25.6%, 27.0%, 21.8% and 7.7%) respectively. A large percentage of the *S. aureus* isolates (25.6% and 27.0%) were from CCP2 and CCP3. All strains were resistant to Penicillin G (PG) (10 μg), Ampicillin (AP) (10 μg), Amoxicillin-Clavulanic acid (AC) (30 μg), Ciprofloxacin (CIP) (5 μg), Erythromycin (E) (15 μg), Ceftriaxone (CRO) (30 μg), Trimethoprime-Sulfamethoxazole (TMP-SMZ) (25 μg) Oxacillin (Ox) (1 μg) and Vancomycin (V) (30 μg), 67.9%, 70.9%, 30.9%, 0%, 32.1%, 23.1%, 7.7%, 60.3% and 38.5% respectively.

**Conclusion:**

The proportion of isolates resistant to CIP, TMP-SMZ, CRO, AC, E and V were low compared to AP, PG and Ox. *S. aureus* is normally resident in humans; therefore, the *S. aureus* present in the cow’s milk may have resulted from transmission between the two species, emphasizing the need to improve sanitary conditions in the milking environment.

## Introduction

*Staphylococcus aureus* is a ubiquitous human pathogen and a common cause of invasive and life threatening infections. It is the most common cause of community-associated cellulitis [1,2] endocarditis [3], and is a common cause of bacteremia [1,4,5]. *S. aureus* strains were once nearly uniformly susceptible to semi-synthetic penicillinase-resistant β-lactams (e.g. methicillin, oxacillin), the most commonly used class of antibiotics for skin infection. These strains were termed ‘methicillin resistant *Staphylococcus aureus,* or MRSA, a term that implied cross-resistance to all β-lactams including all penicillins and cephalosporins.

Staphylococci are normal inhabitants of the skin and mucous membranes of animals and humans. Pathogenic strains are usually coagulase-positive [6] and have been found to cause disease in their hosts throughout the world [7,8]. Diseases in cattle caused by *Staphylococcus aureus* range from simple abscesses and mastitis to the more severe toxic shock syndrome [7-9]. Milk is an excellent growth medium for a large number of micro-organisms, including *S. aureus*[10]. Bacterial contamination of milk usually occurs during the milking process and this depends on the sanitary condition of the environment, utensils used for milking and the milking personnel. It could also result from micro-organisms that enter the udder through the teat opening canal [10].

Antibiotic-resistant *S. aureus* isolates pose a severe challenge to both veterinary and health professions and dairy cattle producers because they have a negative impact on therapy [11,12]. The usage of antibiotics correlates with the emergence and maintenance of antibiotic-resistant traits within pathogenic strains [13]. These traits are coded for by particular genes that may be carried on the bacterial chromosome, plasmids, transposons or on gene cassettes that are incorporated into integrons [14], thus are easily transferred among isolates. Multiple antibiotic resistant *S. aureus* strains have been isolated from milk obtained from cattle, beef and human samples in many parts of the world [13,15-18]. The prevalence of antibiotic resistance usually varies between isolates from the different sampled stations and even between isolates from different herds on the same farm [16].

Determination of levels of *S. aureus* and an evaluation of the antibiotic-resistant phenotypes of the isolates could serve as a tool for determining the hygiene standards implemented during milking. Data on antibiotic resistance could also be used to characterize these opportunistic pathogens, which may further limit the risks associated with the consumption of contaminated milk and its products [19]. The aim of this study was to isolate *S. aureus* from milk obtained from farm level collected milk and further characterization of their susceptibility patterns to nine selected antibiotics.

## Methods and materials

### Area of the study and collection of samples

A cross-sectional study design was used to determine the bacteriological analysis of cow milk in Hawassa town from August 2011-January 2012. A total of 160 milk samples (five samples per family from different HACCP levels) were collected. The study on antibiotic resistance of *S. aureus* isolation was included 32 families of the farms. All of these farms were Hybrid Holland breed herd with average age of 7 years old. An average herd size of the farm was 10 in number and a milking technique of the study community was manual. These farms are situated in the Southern part of Ethiopia at 250 km far apart from the capital city of the country.

Samples of approximately 10 mL of fresh milk were collected from different critical control points (CCP1-CCP5) using sterile sample collection bottles. The samples were immediately transported on ice to the Medical Microbiology Laboratory of the Hawassa University – Referral Hospital for analysis. Upon arrival in the Laboratory, samples were analyzed immediately.

### Isolation of presumptive *S. aureus* from milk samples

Ten-fold serial dilutions were performed using 2% peptone water and aliquots of 100 μL from each dilution were spread plated onto mannitol salt agar (MSA) (Supplied by Oxoid Company). The plates were incubated aerobically at 37°C for 18 h – 24 h. Consequently, the 78 characteristic *S. aureus* colonies that were yellow in color from each MSA plate were further purified by sub-culturing onto MSA plates and the plates were incubated aerobically at 37°C for 18 h–24 h. These isolates were retained for further bacterial identification.

### Bacterial identification

Gram staining was performed [20] and Gram-positive cocci that occurred in clusters under the microscope were subjected to preliminary biochemical tests (the catalase and oxidase tests). The identities of the isolates were confirmed based on positive results for the DNase test, beta-haemolytic patterns on blood agar enriched with 5% (v/v) sheep blood and the coagulase slide test for *S. aureus* using the (PROLD Diagnostics, Canada). The slide agglutination test was performed according to the manufacturer’s instructions. Briefly, cells from a pure colony were placed on the clean area of the slide using a sterile toothpick and a drop of the PROLD reagent was added. These were mixed using the toothpick and the isolates were identified based on the formation of agglutination. An isolates that formed agglutination were recorded as *S. aureus* and maintained at 4°C in 30% glycerol for further characterization by antibiotic susceptibility testing.

### Antibiotic susceptibility

Antibiotic susceptibility tests were performed on all *S. aureus* isolates to determine their antibiotic-resistance profiles [21]. Fresh overnight cultures were prepared and used for antibiotic sensitivity tests. An aliquot (100 μL) from each isolate suspension was spread plated on Mueller Hinton agar (supplied by Oxoid Company). Susceptibilities of the isolates to a panel of nine different antibiotic discs (6 μm in diameter, Mast group LTD MERSEY SIDE, UK) were determined. The antibiotics tested in this study were mostly developed resistant by bacteria as documented in South Africa [22,23]. Antibiotic discs were gently pressed onto the inoculated Mueller Hinton agar to ensure intimate contact with the surface and the plates were incubated aerobically at 37°C for 18 h – 24 h [24]. Inhibition zone diameters were measured and values obtained from the National Committee on Clinical Laboratory Standards [24] were used to interpret the results obtained. *S. aureus* isolates were then classified as resistant, intermediate resistant or susceptible to a particular antibiotic. Multiple antibiotic resistant (MAR) phenotypes were recorded for isolates showing resistance to three and more antibiotics [25].

## Results

### Prevalence of *S. aureus* in tested milk samples

A total of 160 milk samples (32 per HACCP level) were analyzed and were 78 positive for *S. aureus* (Table 1). A total of 210 potential isolates were sub-cultured and further analyzed. However, only 78 isolates satisfied all the identification criteria and were used for subsequent analysis. These constituted a total of 78 *S. aureus* isolates (Table 1). *S. aureus* was obtained from each of the 32 different critical control point (CCP) samples taken per HACCP level, giving a prevalence of *S. aureus* of 48.75% for the 160 samples. The results demonstrated the presence of *S. aureus* in all the milk samples, regardless of the farm setting. However, the levels of contamination with *S. aureus* were higher in milk samples obtained in this study area (Table 1).

**Table 1:** **Details of the *S. aureus *isolates obtained from Hawassa area, South Ethiopia**

**Sample source**	**HACCP level**	**# of sample Collected**	**% of *S. aureus *isolates (level of contamination) with *S. aureus *per HACCP level)**^**a**^
Teat	CCP1	32	14 (17.9%)
Bucket at farm level	CCP2	32	20 (25.7%)
From storage containers at milk collection center	CCP3	32	21 (26.9%)
From transportation container	CCP4	32	17 (21.8%)
After cooling at the pasteurization plant	CCP5	32	6 (7.7%)
			100%

^a^ Percentages were calculated from a total of 78 bacterial isolates studied using the biochemical identification method to determine those positive for *S. aureus*.

### Antibiotic susceptibility

All 78 *S. aureus* isolates were subjected to antibiotic susceptibility tests. Nine antimicrobial agents, from different antibiotic classes were used. Some were selected because some studies have shown that large numbers of bacteria were resistant to them [22,23]. Antibiotics of veterinary and human health relevance were also considered. A summary of the percentage of *S. aureus* that were resistant to these antibiotics is provided in Table 2.

**Table 2:** **Antibiotic-resistance profiles of *Staphylococcus aureus *isolated from milk originating from Hawassa area, South Ethiopia (n = 78)**

**Sample source of milk**	**Antibiotic resistance (%)**								
	**PG**	**AP**	**AC**	**CIP**	**E**	**CRO**	**TMP**	**Ox**	**VA**
Teat	64.3	64.3	14.3	0.0	21.4	14.3	7.1	57.1	57.1
Bucket at farm level	35.0	35.0	10.0	0.0	10.0	5.0	0.0	15.0	70.0
From storage containers at milk collection center	71.4	71.4	14.3	0.0	19.0	9.5	0.0	66.7	57.1
From transportation container	100	100	94.1	0.0	88.2	76.5	23.5	100	23.5
After cooling at the pasteurization plant	83.3	83.3	0.0	0.0	0.0	0.0	0.0	83.3	50.0

**Note**: Percentages were calculated by dividing the number confirmed as *S. aureus* resistant in a particular sample source by the total number of isolates tested.

A large proportion of the isolates of this study area were resistant to Ampicillin (10 μg), Penicillin G (10 μg), and Oxacillin (1 μg), while a similarly large proportion of those from the transportation container were resistant to, Ampicillin (10 μg), penicillin G (10 μg), Erythromycin, (15 μg), Ceftriaxone (10 μg), Amoxicillin-Clavulanic Acid (30 μg), and Oxacillin (1 μg). There were no resistant groups for Ciprofloxacin (5 μg) antibiotics.

In general observation the large percentage of Penicillin G (10 μg), Ampicillin (10 μg) and Oxacillin (1 μg) resistant *S. aureus* were isolated from the study area. These were also resistant to several other antibiotics. Therefore, one can easily conclude that these are Methicillin resistant *S. aureus* (MRSA).

Only a small proportion of the isolates from the teat, bucket and storage container were resistant to Trimethoprime-Sulfamethoxazole (25 μg); Erythromycin (15 μg); Ceftriaxone (30 μg) and Amoxicillin-Clavulanic Acid (30 μg). None of the isolates from bucket, storage container and freshly pasteurized milk were resistant to TMP-SMX (25 μg). Furthermore, 23.5-70% of the isolates from the sample were resistant to Vancomycin (30 μg), while 15-100% of those isolated was resistant to Oxacillin (1 μg).

### MAR phenotypes of *S. aureus*

Multiple antibiotic resistance (MAR) phenotypes were determined for *S. aureus* (Table 3). The predominant MAR phenotypes for *S. aureus* isolated from this study area were PG-AP-AC-E-CRO-Ox and PG-AP-Ox in 19.2% and 20.5% of the isolates, respectively. Furthermore, MAR phenotypes PG-AP-AC-TMX-Ox-VA, PG-AP-AC-E-TMX-Ox-VA and PG-AP-TMX-Ox were obtained in 1.3% of the isolates. The MAR phenotypes PG-AP-AC-E-TMX-Ox, PG-AP-AC-E-CRO-Ox-VA and PG-AP-E were obtained in 1.3% of the isolates. Also PG-AP-AC-E-Ox-VA 3.8% and PG-AP-Ox-VA 7.7% were the MAR phenotypes for *S. aureus* isolated from this study area (Table 3).

**Table 3:** **The predominant multiple antibiotic resistant phenotypes for *Staphylococcus aureus *isolated from cow’s milk obtained from Hawassa area farms, South Ethiopia (n** ***=*** **78)**

**Phenotype**	**Number observed**	**Percentage**
PG-AP-E	2	2.7
PG-AP-Ox	16	20.5
PG-AP-Ox-VA	6	7.7
PG-AP-TMX-Ox	1	1.3
PG-AP-AC-E-Ox-VA	3	3.8
PG-AP-AC-E-CRO-Ox	15	19.2
PG-AP-AC-E-CRO-Ox-VA	2	2.7
PG-AP-AC-E-TMX-Ox-VA	1	1.3
PG-AP-AC-E-TMX-Ox	2	2.7
PG-AP-AC-TMX-Ox-VA	1	1.3

**Note:** The percentage representations of the phenotypes were obtained by dividing the number of a particular phenotype by the total number of multiple antibiotic resistant isolates identified in a given area. *VA*, Vancomycin; *AP*, Ampicillin; *PG*, Penicillin G; *TMP-SMX*, Trimethoprime-Sulfamethoxazole; *E*, Erythromycin; *Ox*, Oxacillin ; *CRO*, Ceftriaxone; *CIP*, Ciprofloxacin; *AC*, Amoxicillin-Clavulanic Acid.

It is thus evident that MAR *S. aureus* was isolated from all CCP. However, among the isolates from this study area 62.8% of the isolates develop MAR. Among all MAR phenotypes of *S. aureus*, 42.9% of them were resistance to six different antibiotics and 6.1% were resistance to seven antibiotics. Fifty one percent (51%) of them were resistance to 3 or 4 antibiotics.

## Discussion

In this study we described the isolation and antibiotic susceptibility characterization of *S. aureus* from milk obtained from five different CCP levels. Our results indicate that 40.6% of the samples were positive for *S. aureus* and that more of these bacteria were positively identified from milk samples. Several studies have been conducted in South Africa to evaluate the prevalence of *S. aureus* in milk [13,26,27]. The results reported in our study were similarly high when compared to those studies [13,27]. Although the prevalence of *S. aureus* has been reported to vary with the size and geographic region of the area sampled, a high proportion of these bacteria in milk relates to poor hygiene practices. Based on observations made during the collection of samples, we therefore report that improper hygiene and poor farm management practices contributed to the presence of *S. aureus* in the milk, especially in those from the Hawassa area. In this study area milk was obtained from animals by washing their hands and/or the utensils and containers used. In certain cases, untreated groundwater was used to wash the containers that were used for milking. This may have contributed to the high level of *S. aureus* isolated. Improving the hygienic conditions of the milking environment and/or utensils may reduce the prevalence of *S. aureus* in milk and prevent its transmission to humans.

A further objective of the study was to characterize and compare the antibiotic-resistance profiles of *S. aureus* isolated from the study area. The motivation for this was the fact that there were no clear studies conducted in the area. Furthermore, the presence of antibiotic-resistant *S. aureus* has been reported to negatively affect the treatment of its associated infections in humans and animals [12,15,23]. We therefore believe that an investigation of the antibiotic-resistance profiles of these isolates may serve as a tool in assessing both the sanitary conditions employed during milking and the health risks that humans may encounter when infected by antibiotic-resistant strains.

PG (10 μg), AP (10 μg) and Ox (1 μg) were the drugs to which a large proportion of the isolates were resistant (Table 4). As shown in Table 4, between 60.0% and 67.9% of the isolates from all CCP levels were resistant to these three drugs. Also VA (30 μg), AC (30 μg), E (15 μg) and CRO (30 μg) were resistance to *S. aureus* about 38.5%, 32.1%, 30.8% and 23.5% respectively. None of the *S. aureus* were resistance to CIP (5 μg). However, very small proportions of *S. aureus* were resistance to TMP-SMX (25 μg) (7.7%). Contrarily to our observations, a study reported that larger percentage (15.7% to 19%) of *S. aureus* isolated was resistant to TMP-SMX (25 μg) [28]. The finding that a large number of *S. aureus* were resistant to PG (10 μg), AP (10 μg) and Ox (1 μg) is, however, a cause for concern and should be further investigated. It is thus our view that the results obtained in this study do not accurately reflect the usage of this antibiotic on farms. We cannot explain this phenomenon.

**Table 4:** **Antibiotic-resistance profiles of *Staphylococcus aureus *from all CCP levels isolated from milk originating from Hawassa area, South Ethiopia (n = 78)**

**Organism**	**Antibiotic resistance (%)**								
	**PG**	**AP**	**AC**	**CIP**	**E**	**CRO**	**TMP**	**Ox**	**VA**
*S. aureus*	67.9	67.9	30.8	0.0	32.1	23.1	7.7	60.3	38.5

**Note:** Percentages were calculated by dividing the number confirmed as *S. aureus* resistant in a particular sample source by the total number of isolates tested.

*VA*, Vancomycin; *AP*, ampicillin; *PG*, penicillin G; *TMP-SMX*, Trimethoprime-Salfamethoxazole; *E*, erythromycin; *Ox*, Oxacillin ; *CRO*, Ceftriaxone; *CIP*, Ciprofloxacin; *AC*, Amoxicillin-Clavulanic Acid.

CIP (5 μg) resistance was almost non-existent in the all CCP levels of the isolates. Also no resistance *S. aureus* strain was isolated from immediate pasteurized milk for AC (30 μg), E (15 μg), CRO (30 μg), TMP-SMX (25 μg) and CIP (5 μg). Despite the fact that a significantly larger number of *S. aureus* isolated from immediate pasteurized milk was resistant to PG (10 μg), AP (10 μg), Ox (1 μg) and V (30 μg), it was evident from our results that this antibiotic is not frequently used in animals by large-scale farmers. Contrarily to our observations, a study reported in South Africa, was 100% of the isolates from the two commercial farms were susceptible to Vancomycin [28]. This drug is no longer used in veterinary medicine in many countries [29] including South Africa, which may account for the results reported here.

Small proportions (67.8%) of the isolates from this study area were resistant to the beta-lactam penicillin G (10 μg), when compared to the isolates from the communal farms, in which 69% to 98% were resistant [28]. The antibiotic resistance obtained for *S. aureus* isolated from does not correlate with the extensive usage of Penicillin on dairy cattle farms in Hawassa town. The Ampicillin-resistance pattern for isolates from these all levels of CCP were similar to the Penicillin G (10 μg) resistance pattern, thus a smaller proportion of the isolates were resistant to this drug when compared to the isolates from the South Africa. Ampicillin (10 μg) is also commonly used on dairy cattle farms in the study area. Resistance to Penicillin G (10 μg) is thus used as a marker to assess the susceptibility of *S. aureus* isolates against other beta-lactam antibiotics [16,29]. This correlates with our findings but suggests that further studies need to be conducted to determine the exact inter-relation between these drugs in the expression of antibiotic-resistant phenotypes on dairy cattle farms in Ethiopia. The Methicillin-resistance pattern had a similar trend to that of several of the previously discussed antibiotics. This is because larger numbers of Oxacillin (10 μg)-resistant isolates were obtained from the milk in this study area.

What is of concern is the large number of Oxacillin (10 μg) (60.3%) that were isolated from the Study area. This is an indicator of MRSA. High levels of MRSA have been identified in patients in the United States and some European countries [30]. In these countries, 44.4%, 34.7%, 41.8% and 32.4% of isolates from patients in the United States, France, Italy and Spain, respectively, were resistant to Methicillin. These levels, however, are lower than those in our study. Methicillin is not used on dairy cattle farms in the South Ethiopia. However, Methicillin resistance could be explained by the inter-relationship between beta-lactam resistance and resistance to this drug. In MRSA, Methicillin resistance is conferred by the Penicillin binding protein (PBP) 2a that is encoded for by the *mec*A gene [31]. PBP2a does not readily bind the beta-lactam moiety. However, in MRSA that are exposed to beta-lactam antibiotics, this PBP2a would contribute to the resistance by providing transpeptidase activity to the native PBPs during cell wall synthesis. In our study, the resistant phenotype PG-AP-Ox was frequently identified usually with the addition of one or more antibiotics (Table 3). It is thus recommended that future studies should confirm the presence of the *mec*A gene in observed MRSA due to these β-lactamase antibiotics.

It has also been documented that MRSA isolates that are resistant to beta-lactam antibiotics may develop induced resistance to Vancomycin (30 μg) [31]. All the isolates from the study area were resistant to Oxacillin (1 μg) were also resistant to penicillin G (10 μg) and Ampicilin (10 μg). In addition, all PG, AP, and AC antibiotic resistance *S. aureus* were also resistance to Ox antibiotics. Moreover, 26.5% of the Oxacillin resistances *S. aureus* were also resistance to Vancomycin (30 μg). We therefore suggest that this may account for the identification of Vancomycin-resistant isolates in the study because Vancomycin is not used in either veterinary or human medicine in the area [29]. Moreover, beta-lactam-induced Vancomycin-resistant MRSA are not easily detected by conventional antibiotic susceptibility tests [32] and this may also account for the very low proportion of Vancomycin-resistant *S. aureus* observed.

The MAR phenotypes (Table 3) obtained in the study correlated with the percentage of antibiotic resistance. Although the development of resistance to a particular antibiotic depends on the level of exposure to the antimicrobials, [14] there are many other factors that are involved. We are therefore suggesting that molecular methods be used to characterize these isolates for the presence of antibiotic-resistance determinants, which may provide data to support our conclusions.

*S. aureus* is normally resident in humans; therefore the *S. aureus* present in the cow’s milk may have resulted from transmission from humans, which raises questions regarding the hygiene practices followed.

## Conclusion and recommendation

*S. aureus* was isolated from all milk samples from Southern Ethiopia. A large proportion of the isolates obtained were resistant to three or more antibiotics. These were also resistant to Vancomycin (30 μg). This was particularly the case in the public setting and is cause for concern. The high level of MAR *S. aureus* and the implications thereof warrant further investigation. One of the aspects that need to be investigated is the cause of the observed resistance phenotypes. Furthermore, impacts and dynamics of genetic antibiotic determinants should also be investigated using molecular methods.

## Competing interests

The authors declare that they have no competing interests.

## Authors’ contribution

DD developed proposal and secured financial support, data collection, laboratory working, analysis of the result, finalizing whole manuscript for publication. SG, read the manuscript and corrected the paper, DY, read the manuscript and laboratory work. All authors read and approved the final manuscript.

## References

[B1] Diekema DJ Pfaller MA Schmitz FJ Survey of infections due to Staphylococcus species: frequency of occurrence and antimicrobial susceptibility of isolates collected in the United States, Canada, Latin America, Europe, and the Western Pacific region for the SENTRY Antimicrobial Surveillance Program, 1997–1999 Clin Infect Dis 2001 32 2 S114 S132 10.1086/320184 11320452

[B2] Brook I Frazier EH Clinical features and aerobic and anaerobic microbiological characteristics of cellulites Arch Surg 1995 130 786 792 10.1001/archsurg.1995.01430070108024 7611872

[B3] Hoen B Alla F Selton-Suty C Changing profile of infective endocarditis: results of a 1-year survey in France JAMA 2002 288 75 81 10.1001/jama.288.1.75 12090865

[B4] Javaloyas M Garcia-Somoza D Gudiol F Epidemiology and prognosis of bacteremia: a 10-years study in a community hospital Scand J Infect Dis 2002 34 436 441 10.1080/00365540110080629 12160171

[B5] Weinstein MP Towns ML Quartey SM The clinical significance of positive blood cultures in the 1990s: a prospective comprehensive evaluation of the microbiology, epidemiology, and outcome of bacteremia and fungemia in adults Clin Infect Dis 1997 24 584 602 9145732 10.1093/clind/24.4.584

[B6] Mahon CR Larsen HS Mahon CR, Manuseseilis G Jr Staphylococcus aureus Textbook of diagnostic microbiology 1995 New York: WB Saunders Company 325 330

[B7] Matsunaga T Kamata S Kakiichi N Uchida K Characteristics of *Staphylococcus aureus* isolated from peracute, acute and chronic bovine mastitis J Vet Med Sci 1993 55 297 300 10.1292/jvms.55.297 8513013

[B8] Larsen HD Sloth KH Elsberg C The dynamics of *Staphylococcus aureus* intramammary infections in nine Danish dairy herds Vet Microbiol 2000 71 89 101 10.1016/S0378-1135(99)00161-3 10665537

[B9] Onasanya A Mignouna HD Thottappilly G Genetic fingerprinting and phylogenetic diversity of *Staphylococcus aureus* isolates from Nigeria Afr J Biotech 2003 2 8 246 250

[B10] Kalsoom F Syed NHS Farzana J Antibiotic resistance pattern against various isolates of *Staphylococcus aureus* from raw milk samples J Res Sci 2004 15 145 151

[B11] Sears PM McCarthy KK Management and treatment of staphylococcal mastitis Vet Clin North Am Food Anim Pract 2003 19 171 185 10.1016/S0749-0720(02)00079-8 12682941

[B12] Brouillette E Malouin F The pathogenesis and control of *Staphylococcus aureus*-induced mastitis: Study models in the mouse Microbes Infect 2005 7 560 568 10.1016/j.micinf.2004.11.008 15777742

[B13] Shitandi A Sternesjö Ǻ Prevalence of multidrug resistant *Staphylococcus aureus* in milk from large and small-scale producers in Kenya J Dairy Sci 2004 87 4145 4149 10.3168/jds.S0022-0302(04)73557-2 15545376

[B14] Rychlik I Gregorova D Hradecka H Distribution and function of plasmids in Salmonella enterica Vet Microbiol 2006 112 1 1 10 10.1016/j.vetmic.2005.10.030 16303262

[B15] Petinaki E Miriagou V Hatzi F Kontos F Maniati M Maniatis AN Bacterial resistance study group. Survey of methicillin-resistant coagulase-negative Staphylococcus aureus in the hospitals of central Greece Int J Antimicrob Agents 2001 18 563 566 10.1016/S0924-8579(01)00454-X 11738345

[B16] Waage S Bjorland J Caugant DA Spread of *Staphylococcus aureus* resistant to penicillin and tetracycline within and between dairy herds Epidemiol Infect 2002 129 193 202 12211588 10.1017/s095026880200715x PMC2869866

[B17] Zhanel GG Hisanaga TL Laing NM Antibiotic resistance in outpatient urinary isolates: Final results from the North American Urinary Tract Infection Collaborative Alliance (NAUTICA) Int J Antimicrob Agents 2005 26 380 388 10.1016/j.ijantimicag.2005.08.003 16243229

[B18] Pesavento G Ducci B Comodo N Lo Nostro A Antimicrobial resistance profile of *Staphylococcus aureus* isolated from raw meat: A research for methicillin resistant *Staphylococcus aureus* (MRSA) Food Cont 2007 18 3 196 200 10.1016/j.foodcont.2005.09.013

[B19] Evenson ML Hinds MW Bernstein RS Bergdoll MS Estimation of human dose of staphylococcal enterotoxin A from large outbreak of staphylococcal food poisoning involving chocolate milk Int J Food Microbiol 1988 7 311 316 10.1016/0168-1605(88)90057-8 3275329

[B20] Cruikshank R Duguid JP Marmoin BP Swain RH Medical microbiology 1975 12 New York: Longman Group Limited 34

[B21] Kirby WMM Bauer AW Sherris JC Turck M Antibiotic susceptibility testing by single disc method Am J Clin Pathol 1966 45 4 5325707

[B22] Ateba CN Bezuidenhout CC Characterisation of Escherichia coli O157 strains from humans, cattle and pigs in the North-West Province, South Africa Int J Food Microbiol 2008 128 181 188 10.1016/j.ijfoodmicro.2008.08.011 18848733

[B23] Moneoang MS Bezuidenhout CC Characterization of enterococci and E. coli isolated from commercial and communal pigs from Mafikeng in the North West Province, South Africa Afr J Microbiol Res 2009 3 3 88 96

[B24] National Committee for Clinical Laboratory Standards (NCCLS) Performance standards for antimicrobial disk and dilution susceptibility tests for bacteria isolated from animals. Approved standard M13-A 1999 Wayne: NCCL

[B25] Rota C Yanguela J Blanco D Carraminana JJ Arino A Herrera A High prevalence of multiple resistances to antibiotics in 144 Listeria isolates from Spanish dairy and meat products J Food Prot 1996 59 938 943 10.4315/0362-028X-59.9.938 31159096

[B26] Lee HJ Methicillin (Oxacillin)-resistant *Staphylococcus aureus* strains isolated from major food animals and their potential transmission to humans Appl Environ Microbiol 2003 69 6489 6494 10.1128/AEM.69.11.6489-6494.2003 14602604 PMC262320

[B27] Gündoğan N Citak S Turan E Slime production, DNase activity and antibiotic resistance of *Staphylococcus aureus* isolated from raw milk, pasteurized milk and ice cream samples Food Cont 2006 17 389 392 10.1016/j.foodcont.2005.01.006

[B28] Ateba CN Mbewe M Moneoang MS Bezuidenhout CC Antibiotic-resistant *Staphylococcus aureus* isolated from milk in the Mafikeng Area, North West province, South Africa S Afr J Sci 2010 106 11/12 243

[B29] Pace JL Yang G Glycopeptides: Update on an old successful antibiotic class Biochem Pharm 2006 71 968 980 10.1016/j.bcp.2005.12.005 16412985

[B30] Mark EJ Karlowsky JA Draghi DC Thornsberry C Sahm DF Nathwani D Epidemiology and antibiotic susceptibility of bacteria causing skin and soft tissue infections in the USA and Europe: A guide to appropriate antimicrobial therapy Int J Antimicrob Agents 2003 22 406 419 10.1016/S0924-8579(03)00154-7 14522104

[B31] Gündoğan N Citak S Yucel N Devren A A note on the incidence and antibiotic resistance of Staphylococcus aureus isolated from meat and chicken samples Meat Sci 2005 69 807 810 10.1016/j.meatsci.2004.10.011 22063160

[B32] Hideaki H Yoshio Y Syuichi N Isao H Ariaki N Keisuke S Method for detecting beta-lactam antibiotic induced Vancomycin resistant MRSA (BIVR) Int J Antimicrob Agents 2004 23 1 5 14732306 10.1016/j.ijantimicag.2003.05.018

